# Cascaded Hough Transform-Based Hair Mask Generation and Harmonic Inpainting for Automated Hair Removal from Dermoscopy Images

**DOI:** 10.3390/diagnostics12123040

**Published:** 2022-12-04

**Authors:** Amira S. Ashour, Basant S. Abd El-Wahab, Maram A. Wahba, Diaa-Eldin A. Mansour, Abeer Abd Elhakam Hodeib, Rasha Abd El-Ghany Khedr, Ghada F. R. Hassan

**Affiliations:** 1 Department of Electronics and Electrical Communications Engineering, Faculty of Engineering, Tanta University, Tanta 31511, Egypt; 2 Department of Electrical Power and Machines Engineering, Faculty of Engineering, Tanta University, Tanta 31511, Egypt; 3 Department of Dermatology and Venereology, Faculty of Medicine, Tanta University, Tanta 31511, Egypt; 4 Department of Clinical Oncology, Faculty of Medicine, Tanta University, Tanta 31511, Egypt

**Keywords:** skin cancer, dermoscopy, hair mask generation, Hough transform, harmonic image inpainting, adaptive Wiener filter

## Abstract

Restoring information obstructed by hair is one of the main issues for the accurate analysis and segmentation of skin images. For retrieving pixels obstructed by hair, the proposed system converts dermoscopy images into the L*a*b* color space, then principal component analysis (PCA) is applied to produce grayscale images. Afterward, the contrast-limited adaptive histogram equalization (CLAHE) and the average filter are implemented to enhance the grayscale image. Subsequently, the binary image is generated using the iterative thresholding method. After that, the Hough transform (HT) is applied to each image block to generate the hair mask. Finally, the hair pixels are removed by harmonic inpainting. The performance of the proposed automated hair removal was evaluated by applying the proposed system to the International Skin Imaging Collaboration (ISIC) dermoscopy dataset as well as to clinical images. Six performance evaluation metrics were measured, namely the mean squared error (MSE), the peak signal-to-noise ratio (PSNR), the signal-to-noise ratio (SNR), the structural similarity index (SSIM), the universal quality image index (UQI), and the correlation (C). Using the clinical dataset, the system achieved MSE, PSNR, SNR, SSIM, UQI, and C values of 34.7957, 66.98, 42.39, 0.9813, 0.9801, and 0.9985, respectively. The results demonstrated that the proposed system could satisfy the medical diagnostic requirements and achieve the best performance compared to the state-of-art.

## 1. Introduction

Dermoscopy, also known as dermatoscopy, is a non-invasive skin imaging technique that enhances the visibility of the subsurface structures compared to typical clinical images. Close examination of pigmented skin lesions in this manner improves the accuracy of the clinical diagnosis by introducing new morphological indicators for recognizing malignant lesions apart from other melanocytic and non-melanocytic benign skin lesions. However, using dermoscopy by inexperienced physicians affects the diagnosis accuracy. Therefore, the development of computer-based automated diagnostic systems is of significant importance in the early diagnosis of malignant lesions.

The existence of hair in dermoscopic images is a major challenge in computer-aided diagnostic systems. Hair pixels usually occlude the lesion area affecting its morphological characteristics, such as texture and boundary. Hence, hair removal is an essential pre-processing stage in such systems. Inefficient hair removal procedures from dermoscopy images cause poor segmentation and inaccurate pattern recognition. The conventional process of hair removal includes two main steps, namely (i) detecting and removing the hair pixels, then (ii) estimating the texture and color of the lesion and/or skin behind the identified hairs and reconstructing the removed hair pixels by the estimated pixels of skin. The main challenges of hair detection are the presence of thick and thin hairs along with the presence of hairs with colors that are close to the skin lesion colors. Moreover, the reconstruction techniques may disrupt the tumors’ texture and patterns by causing blurring or color bleeding. Furthermore, most hair removal systems require complex computations and high processing time.

Several techniques have been proposed for hair removal in dermoscopy images. One of the most widely implemented techniques is DullRazor [1], which applies thresholding in the three color bands after applying morphological closing operations in different directions. Then, the verified hair pixel is replaced by applying bilinear interpolation, followed by applying a median filter. Zagrouba et al. [2] introduced DullRazor and a 5 × 5 median filter to exclude thick and thin hair, which was applied to 200 RGB images and achieved 79.1% classification accuracy for malignant and benign lesions. Fiorese et al. [3] drew the hair on 20 RGB images and introduced top hat filtering to obtain the hair mask. After that, thresholding was applied to verify if the output of the hair mask represented true or false hair based on the geometric shape, followed by partial differential equation (PDE)-inpainting. This method resulted in 15.6% misclassification compared to the DullRazor which achieved 47.1%. Kiani et al. [4] applied edge detection using the Prewitt filter and radon transform to create the correct hair mask. Then, the non-hair pixels were removed and the hair pixels were replaced by averaging the gray levels of the neighbor background.

Moreover, Xie et al. [5] designed a system based on morphological closing-based top hat filtering and thresholding for hair detection. Additionally, PDE-based inpainting anisotropic diffusion was utilized to replace the hair pixels. This procedure was employed on 40 images in which the hair masks were drawn manually as ground truth resulting in 18% hair extraction error compared to 30.7% using DullRazor. For hair detection, Abbas et al. [6] applied two-dimensional derivatives of Gaussian (DOG) and proposed an exemplar-based inpainting method for hair replacement. A region-based active contour model was used for the segmentation process. This method was applied on 320 images and improved the true detection rate by 4.31%. Abbas et al. [7] used the derivative of Gaussian (DOG) and morphological techniques for hair detection in the CIE L*a*b* color space, followed by coherence transport inpainting. This method was applied on 100 images and achieved 2.98% hair detection error (HDE) and 4.21% tumor-disturb patterns (TDP). Huang et al. [8] applied a multiscale matched filter with hysteresis thresholding for hair detection. Additionally, a region growing procedure with linear discriminant analysis (LDA) was employed for recovering complicated hair intersection patterns. This procedure was applied on 20 images and resulted in a 58% hair detection rate. Toossi et al. [9] utilized an adaptive canny edge detector with a morphological operator for hair detection, in addition to coherence transport inpainting with multiple resolutions on 50 images. The results achieved 88.3% diagnostic accuracy, a 93.2% true detection rate (TDR), and a 4% false positive rate (FPR). Joanna et al. [10] applied a Laplacian filter and top-hat transform on 50 dermoscopy images leading to 88.7% diagnostic accuracy and 90.8% sensitivity.

Francisco et al. [11] applied the histogram to enhance the contrast and a Canny edge detector for hair detection. George et al. [12] exploited the grayscale morphological closing with various direction structures on the red channel only, followed by Otsu’s thresholding, then 2D interpolation to restore the hair pixels. Koehoorn et al. [13] utilized gap-detection using multiscale skeletons and a fast marching process for hair pixel inpainting. Salido et al. [14] applied a median filter and bottom-hat filter on each of the RGB color channels for hair detection followed by morphological operation, in addition to replacing hair pixels using harmonic inpainting. This procedure achieved a 33.41 peak signal-to-noise ratio (PSNR); on the other hand, the DullRazor achieved 32.44. Hamet et al. [15] introduced a curvilinear hair detector based on a color morphological process over the CIE L*a*b* space followed by a morphological inpainting procedure. Zaqout et al. [16] utilized a top-hat operator for finding the hair structures using YIQ space, then the histogram and morphological closing operation were utilized on each block for inpainting. Bibiloni et al. [17] introduced soft color closing top-hat operators for hair detection, while morphological transformations were used for the inpainting process based on different sized kernels of 9 × 9 and 11 × 11. Finally, Talavera–Martínez et al. [18] designed convolutional neural networks for the hair removal process. The results showed superior performance compared to other state-of-the-art methods in terms of the average values of the different metrics, namely 27.847 MSE, 0.926 SSIM, 35.137 PSNR, and 4.790 RMSE using 185 dermoscopy images.

The limitation of the previous related work is extracting and removing the hair pixels without affecting the region of interest (ROI). Accordingly, in the proposed technique, the Hough transform block was applied to overcome this limitation. As the hair does not usually take a specific shape, such as a curve or a line, the image was broken into blocks to approximate the shape of the hair into a line or a small curve. Subsequently, the Hough transform (HT) was applied on each block to produce a sub-hair mask from each one. Then, the sub-hair masks were combined to produce the overall hair mask of the original image.

## 2. Materials and Methods

A total of 900 dermoscopy images from the International Skin Imaging Collaboration (ISIC) 2016 challenge dataset [19] were used to evaluate the proposed hair removal technique. The proposed hair removal system depends on the iterative-based thresholding process as follows: (i) the input image is converted into a grayscale image using principal component analysis (PCA); (ii) the binary image is generated by thresholding the resultant gray image; (iii) the hair mask is created by splitting the binary image into blocks and applying Hough transform (HT) on each block; finally, (iv) harmonic inpainting is applied on the product of the original image and the hair mask.

### 2.1. Image Preprocessing

The input RGB dermoscopy image was transformed to the L*a*b* color space. Subsequently, the L*a*b* color space was converted into a grayscale image by applying PCA. The contrast of the enhanced grayscale image was adjusted by contrast-limited adaptive histogram equalization (CLAHE). To separate the background pixels, the average filter was applied to background exclusion. After that, the thresholding method was employed to convert the grayscale image into a binary image.

### 2.2. Iterative Thresholding Method

The thresholding process using the iterative procedure in [20] was applied to the grayscale images to obtain their corresponding binary images. Initially, the histogram was fragmented into two parts by utilizing an initial threshold value. Two mean values were computed from the foreground and the background gray pixel values. Then, the average value of the two computed means was computed to obtain the new threshold as follows:

(1) $$Th\left(i\right)=\frac{Th_{a}+Th_{b}}{2}$$

where $Th_{a}$ and $Th_{b}$ are the mean value of the above part and the mean value of the below part, respectively. This iterative procedure was repeated until the threshold value became fixed. These steps are presented as follows in Algorithm 1.

**Algorithm 1.** Thresholding procedure***Start***   *i=0* ***Input*** gray image     ***If***
*i*=0        $Th\left(i-1\right)$= Initial value     *End*
***Fragment*** the histogram utilizing $Th\left(i-1\right)$ into two parts ***Compute*** the mean value of above $Th_{a}$ and the mean value of below $Th_{b}$
***Compute*** the new threshold value as in Equation (1)     ***If*** $Th\left(i\right)\neq Th\left(i-1\right)$       *i=i+1*       $Th\left(i-1\right)$=$Th\left(i\right)$    *Repeat* from start    ***Else if***
       Th=$Th\left(i\right)$    ***Normalize*** the Th to the range [0, 1]    ***End if***
***Output*** Th ***End***


The estimated threshold was applied for thresholding the gray image to obtain the binary image. Subsequently, the binary image was exploited in the hair mask detection process, as the binary image was then divided into blocks, and HT was applied on each block to extract the line or the curve representing the hair.

### 2.3. Hough Transform

Hough transform [21,22] is a technique for extracting the edges from the images. The HT is based on mapping a point in the image space into a line or a curve in the Hough space. Subsequently, applying some of the Hough space properties, identifying and detecting pixel clusters having the same properties indicate that they belong to the same line or group of lines. The required information to draw the detected lines was provided. Mathematically, a line or an edge can be represented in the image space using the following formula [23]:

(2) y=ax+b

where *a* and *b* are the slope of the line and the intercept of the line with the *y*-axis, respectively. The HT is based on representing a line or edge in the polar space. So, each point in an image can be transformed into a sinusoidal curve in HT using the following transformation [24]:

(3) x=rsinθ

(4) y=rcosθ

(5) ρ=xcosθ+ysinθ

where *ρ* represents the distance from the origin to the closest point on the straight line, and *ϴ* is the angle between the *x*-axis and the line connecting the origin with that closest point, as shown by the following equations.

(6) $$r=\sqrt{x^{2}+y^{2}}$$

(7) $$\theta =\tan^{-1}\left(\frac{x}{y}\right)$$

Accordingly, the HT maps each (*x*, *y*) position in the image into a sinusoidal curve in the Hough space (ρ,θ). Accordingly, the resultant binary image *b* (with m×n) from the thresholding process containing hair was divided into blocks *b_i_* (with q×q) to approximate the hair into a line or a small curve. After that, the HT was applied on each block to track the hairs and wipe the pixels that do not belong to the hair, which preserves the region of interest without any distortion. This process can be illustrated as follows:

(8) $$b_{i}=\frac{b}{N}$$

(9) $$b=\left(\begin{array}{ccc} b_{1} & \ldots & b_{i}\\ \vdots & \ddots & \vdots \\ b_{i} & \cdots & b_{N} \end{array}\right)$$

(10) $$B_{i}=HT\left(b_{i}\right)$$

(11) $$B=\left(\begin{array}{ccc} B_{1} & \ldots & B_{i}\\ \vdots & \ddots & \vdots \\ B_{i} & \cdots & B_{N} \end{array}\right)$$

where b, $b_{i}$, and N are the binary image, binary block, and the number of blocks, respectively. HT was applied on each block *b_i_* to obtain the corresponding hair sub-mask $B_{i}$. The overall mask B was obtained by combining the resultant hair mask blocks. Next, the overall hair mask was multiplied by the original RGB image to remove the hair pixels. The value at the removed hair pixel position was restored using a harmonic inpainting procedure.

### 2.4. Harmonic Inpainting

Image inpainting [25,26,27] is used for area restoration by restoring a missing region in an image. It is usually obtained by using the known information provided by the present regions. In the present case, the product of the resultant hair mask and the dermoscopy image have the missing segments. Accordingly, the information covered by the hair pixels is then retrieved by applying a harmonic inpainting procedure. The harmonic inpainting was applied on each of the RGB color channels. The missing segment in the inpainting domain can be filled using the regularizing term $\psi \left(u\right)$ as follows [28]:

(12) minu∫ΩDu−u02dx+ψu

where *u*_0_, *u* and $\psi \left(u\right)$ are known information, missing segment, and the regularizing term, respectively. The fidelity term was utilized to minimize Equation (11) performed in a suitable Banach space S, which relies on the election of the regularizing function. In the event of the harmonic inpainting, the regularizing term $\psi \left(u\right)$ can be represented as follows [29]:

(13) $$\psi \left(u\right)=\alpha \int_{\Omega }{\left|\nabla u\right|}^{2}dx$$

(14) $$S=W^{1,2}\left(\Omega \right)$$

### 2.5. The Proposed Hair Removal Method

**Figure 1:**
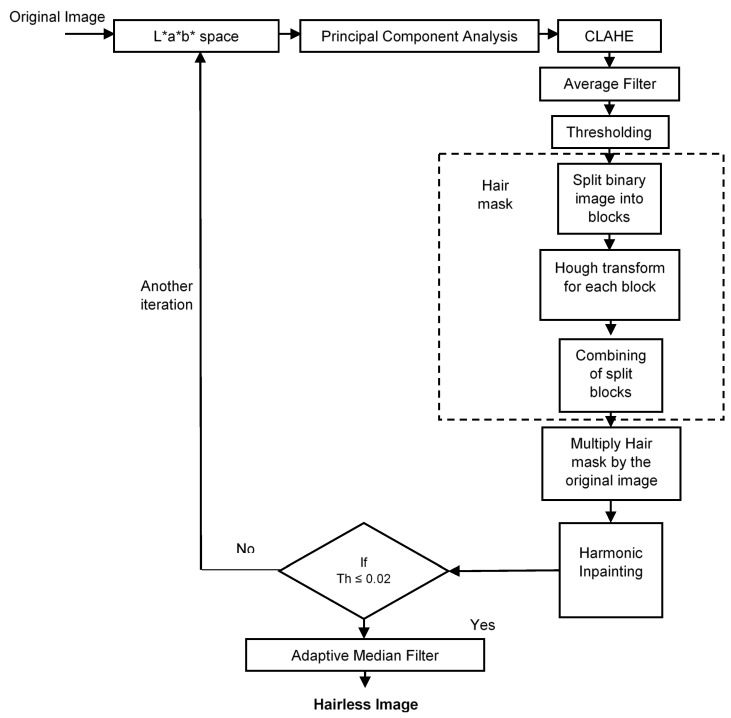
Proposed hair removal technique.

The proposed hair removal system is based on the iterative method outlined in Figure 1. The input RGB dermoscopy image is transformed into the CIE L*a*b* color space. The PCA is then applied to convert the L*a*b* image into an enhanced grayscale image, followed by CLAHE to improve the contrast of the grayscale image. Then, the average filter is employed for background alienation.

The resultant gray image is transformed into a binary image by computing a global threshold that can be applied to convert an intensity image to a binary image, and then the connected components (objects) that are less than 25 pixels are removed from the binary image. Successively, the hair mask is generated by splitting the binary image into blocks to approximate the hair into a line or curve to detect the hair by using HT. The output hair mask is multiplied by the RGB original image, and then the harmonic inpainting is applied to remove the hair pixels. To ensure that all the hair pixels in the image are removed, the threshold is computed again. If the threshold is greater than 0.02 (after trial and error), all the pre-mentioned steps are repeated until the threshold becomes less than 0.02. Finally, the adaptive median filter, median filter 3 × 3, and median filter 5 × 5 are applied separately to evaluate their impact on smoothing the output hairless image.

## 3. Results and Discussion

### 3.1. Implementation of the Proposed Technique

**Figure 2:**
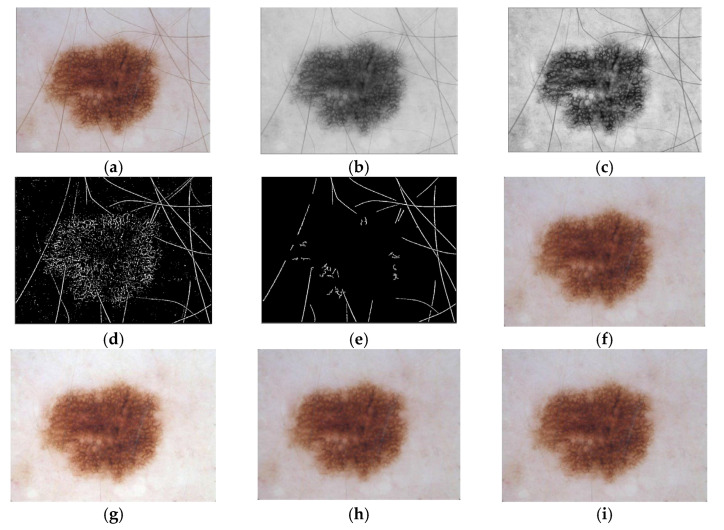
Qualitative analysis of the proposed hair removal system, where (**a**) original image; (**b**) the output gray image from PCA; (**c**) enhanced gray image; (**d**) binary image after thresholding; (**e**) collected mask from all iterations; (**f**) hairless image; (**g**) adaptive median filter; (**h**) 5 × 5 median filter; (**i**) 3 × 3 median filter.

According to the phases of the proposed hair removal system, at every iteration, the PCA was applied to obtain the grayscale image from the L*a*b* color space dermoscopy image, as revealed in Figure 2a,b. The resultant gray image was enhanced utilizing CLAHE, as shown in Figure 2c. The background was alienated by applying (7 × 7) average filter (after trial and error). Afterward, the thresholding method was applied to obtain the binary image, as shown in Figure 2d. The resultant binary image was scanned from left to right and from top to bottom and broken into blocks with the block size 50 × 50 (using trial and error). The block location was kept by saving the first-pixel position, which indicates the minimum row and minimum column. For each data block in the image, the HT was applied on the block to obtain the hair sub-mask. The resultant block from the HT was returned to the saved location to obtain the overall hair mask. Consequently, the overall hair mask for this iteration was obtained by combining the hair mask for each block multiplied by the original image. The harmonic inpainting was then used to remove the hair pixels. After every iteration, depending on the threshold, the overall hair mask and the hairless image were obtained, as shown in Figure 2e,f. Finally, the hairless image was enhanced using an adaptive median filter, 5 × 5 median filter, or 3 × 3 median filter, as reported in Figure 2g–i.

**Figure 3:**
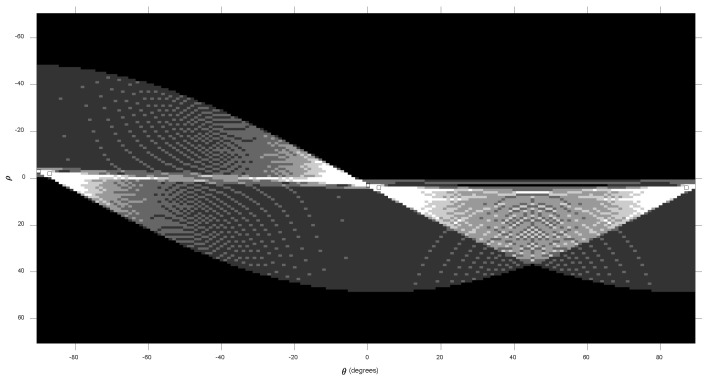
Line representation in Hough space.

**Figure 4:**
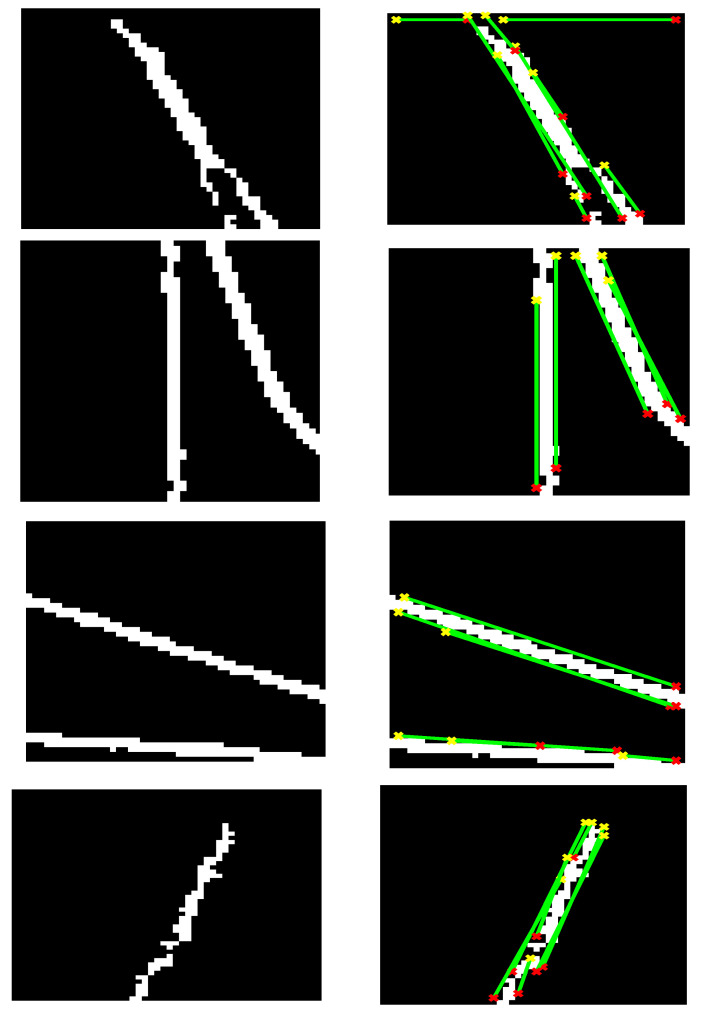
Sub-masks using Hough transform at each block.

At the HT stage, the HT was applied in each binary block to obtain the curve and the line in the image space. It was obtained by converting the block in the image space into the Hough space. In the Hough space, the features of the line and curve can be extracted, as shown in Figure 3. The result from the HT is the hair sub-masks, as illustrated in Figure 4.

Figure 4 illustrates that the HT has tracked the curve or the line in the block. The HT was applied under the conditions using trial and error, where (i) the gap between 2 lines was filled to recover the hair pixels in case the distance was 5 pixels or less, and (ii) the length of the line or the curve less than 5 pixels was considered as noise. The HT results from each block were collected to produce the overall hair mask.

### 3.2. Proposed System Evaluation

The performance of the hair removal system is assessed by measuring six performance measures. These quality metrics are the mean squared error (MSE) [30], the structural similarity index (SSIM) [31], the peak signal-to-noise ratio (PSNR) [31], the signal-to-noise ratio (SNR) [31], the universal quality image index (UQI) [32], and the correlation (C) between the output hairless image and the reference image.

To evaluate the proposed hair removal method, the DullRazor program was applied to all the dataset images in order to obtain the hairless images, which were used as the reference image in the evaluation process, as the DullRazor method is considered the benchmark for hair removal methods in CAD systems. Table 1 compares the performance of the different filters and inpainting techniques in terms of the MSE, PSNR, SNR, SSIM, UQI, and C. The evaluated inpainting techniques include the harmonic, Mumford–Shah, AMLE, Cahn–Hilliard, and transport inpainting [33].

Table 1 demonstrates the superiority of the proposed system with harmonic inpainting and adaptive median filter, where it achieved 58.6188 PSNR while achieving PSNR of 43.5902 and 45.5699 using a 5 × 5 median filter and 3 × 3 median filter, respectively. Also, the SNR value using the adaptive median filter was 33.9071, while the other filters achieved SNR values of 19.5241 and 21.5039. The least MSE was observed for the adaptive median filter, as the MSE value reached 112.59, while the other filters’ MSE values were 424.46 and 309.87. Also, the highest SSIM, UQI, and C values were observed for the adaptive median filter, as these metrics reached 0.9655, 0.9993, and 0.9904, respectively. The harmonic inpainting realized the least processing time of 22.41 s, while the processing time for Mumford–Shah, AMLE, Cahn–Hilliard, and Transport inpainting were 181.33 s, 139.38 s, 336.40 s, and 422.26 s, respectively.

### 3.3. Comparison between the Proposed System and DullRazor Using HairSim

In this section, a study is carried out to compare the performance of the proposed system to the DullRazor procedure. To conduct this comparison, we initially selected the hairless images from the database, which were used as reference or clean images for evaluating the systems. Then, the hair simulation was applied to those clean images. Two different hair simulators were applied. The first implemented simulator was by Attia et al. [34] to produce pragmatic results (we denote it by RH). The other simulator was by Mirzaalian et al. [35,36], whose program is accessible and called “HairSim” (we denote it by HS). Table 2 displays the comparison between DullRazor and the proposed method by applying the two hair simulation programs.

Table 2 reveals that the results of the proposed method are superior to the DullRazor results using the 2 hair simulators achieving PSNR values of 63.3816 (RH) and 66.9707 (HS), while DullRazor achieved PSNR values of 43.2751 (RH) and 45.6519 (HS). Additionally, the SNR values of the proposed method were 29.2967 (RH) and 35.8962 (HS). On the other side, DullRazor achieved SNR values of 19.1835 (RH) and 21.5865 (HS). The least MSE values were established by the proposed method, as the MSE values were 32.9812 (HS) and 25.9761 (RH), while DullRazor MSE values were 194.26 (HS) and 316.0367 (RH). The highest SSIM, UQI, and C were also realized by the proposed method. Therefore, the proposed method is superior to DullRazor for hair removal.

### 3.4. Proposed Method Evaluation on Clinical Images 

In this section, the clinical images were studied to evaluate the effectiveness of the proposed system and its ability to handle clinical, real-time images. The proposed method was applied on 284 clinical images. To evaluate the results, the DullRazor algorithm was initially applied to the clinical images. The resultant images from DullRazor were considered as the reference images in the evaluation. Table 3 reflects the average results of evaluating the proposed method on the clinical images.

**Figure 5:**
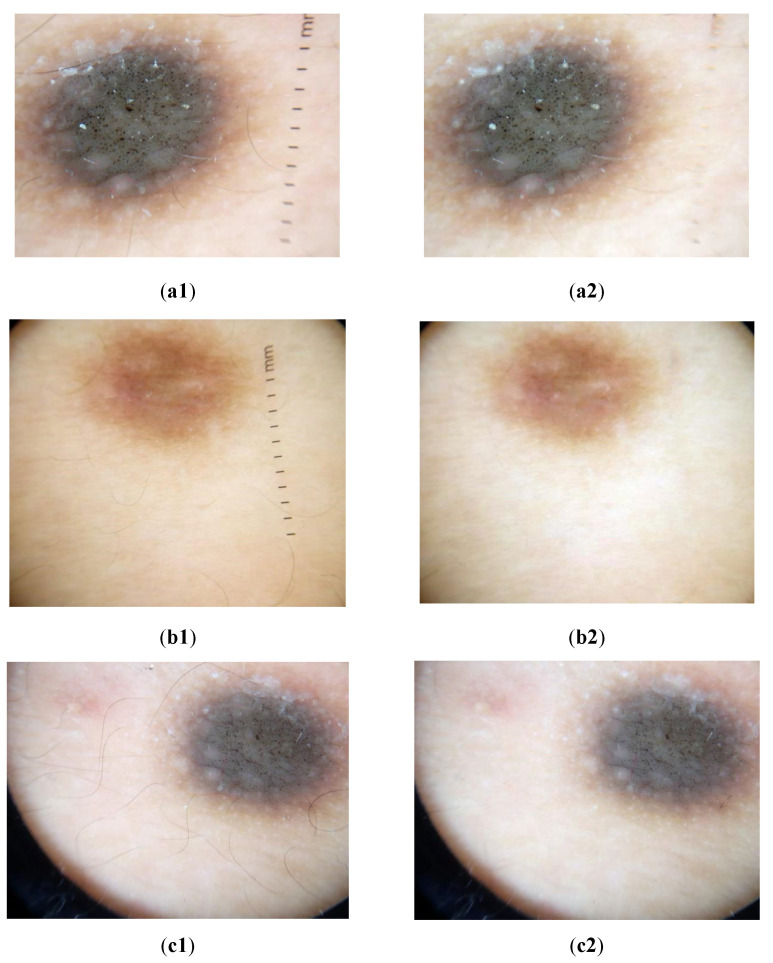
Samples of clinical images before and after applying the proposed hair removal method, where (**a1**–**c1**) images before hair removal and (**a2**–**c2**) images after hair removal method.

Table 3 shows that the proposed method resulted in an MSE value of 34.7957, in addition to achieving MSE, PSNR, SNR, SSIM, UQI, and C values of 34.7957, 66.98, 42.39, 0.9813, 0.9801, and 0.9985, respectively. Samples of the resultant clinical images after applying the proposed method are shown in Figure 5.

### 3.5. Discussion

To further verify the primacy of the proposed method, Table 4 compares the proposed system and six traditional hair removal procedures in terms of their performance. The six methods have applied various hair detection, extraction, and inpainting methods. These methods were chosen on the basis of their accessibility and scalability. These methods are Abbas et al. [7], Huang et al. [8], Bibiloni et al. [17] using 9 × 9 and 11 × 11 kernel filters Toossi et al. [9] and Xie et al. [5], as a comparison with state-of-the-art.

From Table 4, the proposed method achieved the best result compared to the traditional method, in addition to its ability to remove thin and thick hair. The least MSE values were established by the proposed method, as the MSE values were 25.9761 (RH) and 32.9812 (HS). The biggest PSNR, SSIM, and UQI values were also observed for the proposed method, as the PSNR values were 63.3816 (RH) and 66.9707 (HS), the SSIM values were 0.9800 (HS) and 0.9910 (RS), and the UQI values were 0.998 (HS) and 0.999 (RH).

The proposed hair removal system achieved the best performance compared to the other methods. This method applied different sizes of median and adaptive median filters to choose the most suitable for dermoscopy images. The result found that the adaptive median filter was suitable for the normal type of dermoscopy images because it decides and classifies which pixels in the image are affected by noise and replaces these only by the value of the median pixel from the neighboring pixels. On the other side, the adaptive homomorphic, anisotropic diffusion, and Frost filters are suitable for ultrasound images [37,38]. Therefore, in future work, other filters, such as the Wiener filter and Wavelet transform, will be studied to compare them and enhance the overall system performance. 

## 4. Conclusions

This paper has introduced a new system for automated hair removal using Hough transform and harmonic inpainting. This process is of major significance in dermoscopy image pre-processing, as it aids in the accurate classification of skin lesions by occluding the noisy information and obstruction caused by hairs. This proposed method has established superior performance with respect to the traditional methods, as well as its ability to eliminate thin and thick hairs without deteriorating the ROI.

The proposed method was applied to two different datasets, namely the ISIC 2016 dermoscopy dataset and clinical dataset. Firstly, to estimate the system performance, DullRazor was applied to all the images in the datasets to obtain the hairless (i.e., reference) images. The results showed that the proposed system with harmonic inpainting and adaptive median filter achieved the highest results. The system achieved MSE, PSNR, SNR, SSIM, UQI, and C values of 112.59, 58.6188, 33.9071, 0.9655, 0.9993, and 0.9904, respectively, applied to the ISIC 2016 dataset, and MSE, PSNR, SNR, SSIM, UQI, and C values of 34.7957, 66.98, 42.39, 0.9813, 0.9801, and 0.9985, respectively, when applied to the clinical dataset. Moreover, to verify the superiority of the proposed method, hairless images were selected from the dataset to compare the algorithms. Next, the hair simulation was implemented to these specific images. Accordingly, the proposed method achieved the best performance compared to the other algorithms, such that the MSE values were 25.9761 (RH) and 32.9812 (HS). The highest PSNR, SSIM, and UQI values were also observed, as the PSNR values were 63.3816 (RH) and 66.9707 (HS), the SSIM values were 0.9800 (HS) and 0.9910 (RS), and the UQI values were 0.998 (HS) and 0.999 (RH).

## Figures and Tables

**Table 1:** Performance quality metrics of the proposed hair removal with different filters and inpainting techniques.

	Inpainting Technique	MSE	PSNR	SNR	SSIM	UQI	C
Adaptive Median Filter	Harmonic	112.59	58.6188	33.9071	0.9655	0.9993	0.9904
	Mumford–Shah	123.19	57.6181	32.5244	0.9585	0.9743	0.9902
	AMLE	129.99	55.3174	31.2513	0.9566	0.9737	0.9897
	Cahn–Hilliard	6824.3	31.9174	7.85134	0.7747	0.9275	0.7182
	Transport	460.31	49.9265	25.8604	0.9233	0.9797	0.9669
Median Filter (5 × 5)	Harmonic	424.46	43.5902	19.5241	0.9406	0.9597	0.9872
	Mumford–Shah	420.44	43.6119	19.5459	0.9409	0.9997	0.9867
	AMLE	424.40	43.6322	19.5661	0.9413	0.9998	0.9874
	Cahn–Hilliard	25,449.2	24.0742	0.00812	0.6587	0.6069	0.7204
	Transport	840.03	40.3715	16.3054	0.9116	0.9965	0.9629
Median Filter (3 × 3)	Harmonic	309.87	45.5699	21.5039	0.9538	0.9698	0.9888
	Mumford–Shah	305.09	45.6182	21.5522	0.9642	0.9997	0.9882
	AMLE	311.93	45.5933	21.5272	0.9639	0.9997	0.9889
	Cahn–Hilliard	25,451.2	24.0738	0.0078	0.6656	0.6065	0.7175
	Transport	759.55	40.8909	16.8249	0.9287	0.9965	0.9637

**Table 2:** Comparison between the proposed algorithm and DullRazor using both RH and HS hair simulators.

		MSE	PSNR	SNR	SSIM	UQI	C
Proposed Method	HS	32.9812	66.9707	35.8962	0.9800	0.9980	0.9934
	RH	25.9761	63.3816	29.2967	0.9910	0.9990	0.9902
DullRazor	HS	194.26	45.6519	21.5865	0.9184	0.9996	0.9856
	RH	316.0367	43.2751	19.1835	0.8972	0.9968	0.9844

**Table 3:** Evaluation of the proposed method on the clinical images.

	MSE	PSNR	SNR	SSIM	UQI	C
Proposed method	34.7957	66.9868	42.3960	0.9813	0.9801	0.9985

**Table 4:** Comparison of the proposed hair removal system against six conventional hair removal methods.

		Proposed Method	Abbas	Huang	Bibiloni (9 × 9 Kernel)	Bibiloni (11 × 11 Kernel)	Toossi	Xie
**MSE**	HS	32.9812	257.0073	87.0417	123.8135	127.9177	263.8542	47.8494
	RH	25.9761	143.4654	106.9602	100.4868	98.2271	142.2038	36.3482
**SSIM**	HS	0.9800	0.8898	0.9348	0.8898	0.8900	0.8751	0.9599
	RH	0.9910	0.9018	0.8862	0.9245	0.9245	0.8934	0.9531
**PSNR**	HS	66.9707	25.3906	40.3325	34.6192	34.1082	24.6888	53.7967
	RH	63.3816	33.0639	38.0847	39.2326	39.5155	33.1484	48.7572
**UQI**	HS	0.998	0.993	0.997	0.996	0.996	0.993	0.997
	RH	0.999	0.994	0.998	0.996	0.996	0.994	0.999

## Data Availability

Data available at: https://challenge.isic-archive.com/data/ (accessed on 5 January 2021).
